# The Effectiveness of Occlusal Splint Therapy vs Physiotherapy in the Management of Temporomandibular Disorders: A Prospective Comparative Study

**DOI:** 10.7759/cureus.100851

**Published:** 2026-01-05

**Authors:** Amine Chafii, Imane El Bassity, Nezha Ben El Hammi, Asmaa Ait Dambark, El Mehdi Jouhadi

**Affiliations:** 1 Dentistry, Faculty of Dental Medicine, Université Hassan II de Casablanca, Casablanca, MAR; 2 Fixed Prosthodontics, Faculty of Dental Medicine, Université Hassan II de Casablanca, Casablanca, MAR

**Keywords:** disc displacement, occlusal splint therapy, physiotherapy, temporomandibular disorders (tmd), tmd management

## Abstract

Objective

The objective of this study was to evaluate and compare the therapeutic outcomes of occlusal splint therapy versus physiotherapy in patients diagnosed with temporomandibular disorders (TMD) at the *Entre de Consultations et de Traitements Dentaires* (Dental Consultation and Treatment Center), Casablanca, Morocco.

Methods

This prospective longitudinal study included 70 patients diagnosed with TMD over an eight-month period. Participants were treated with either occlusal splints (62.8%) or physiotherapy (37.2%), based on clinical indication and patient preference. Diagnoses were confirmed using clinical criteria, and outcomes were assessed based on symptom resolution, treatment duration, and patient-reported improvement. Statistical analyses included both univariate and bivariate methods, with significance set at p < 0.05.

Results

The sample exhibited a strong female predominance (82.9%) and a mean age of 35.6 ± 14.9 years. The most common symptoms were joint noises (70%), pain (68.6%), and limited mandibular mobility (65.7%). Diagnoses included disc displacement with reduction (52.9%) and without reduction (47.1%). Overall, 84.3% of patients achieved recovery. A significantly higher recovery rate was observed in the splint group (95.5%) compared to the physiotherapy group (65.4%) (p = 0.001). While physiotherapy yielded faster early improvements, splint therapy required fewer clinical visits and offered superior long-term outcomes. No significant differences were found based on age, sex, or type of disc displacement.

Conclusion

Occlusal splint therapy demonstrated greater effectiveness than physiotherapy in managing TMD, achieving significantly higher long-term recovery rates. Nevertheless, physiotherapy represents a valuable and rapid-acting alternative, particularly in early intervention or as part of a combined therapeutic approach. Considering the limitations of this study, such as sample size and follow-up duration, future high-quality, long-term randomized controlled trials are warranted to refine and optimize evidence-based TMD management strategies.

## Introduction

Temporomandibular disorders (TMD) are prevalent conditions causing orofacial pain and functional impairment, affecting approximately 10% of adults. They encompass a heterogeneous group of musculoskeletal dysfunctions involving the temporomandibular joint (TMJ) and masticatory muscles, commonly characterized by joint sounds, musculo‑articular pain, and mandibular movement disorders.​

Due to the multifactorial nature of TMD and the lack of consensus on the optimal management strategy, conservative approaches (behavioral advice, medication, physical therapy/rehabilitation, and other non‑invasive modalities) are generally recommended as first‑line options. Occlusal splint therapy is widely used as a conservative reference treatment; however, it may modify occlusion and sometimes requires subsequent orthodontic or prosthetic adjustment [1,2]. Rehabilitation therapy represents a non‑invasive alternative aimed at improving muscle function, mobility, coordination, and pain control. Nevertheless, comparative evidence between rehabilitation and occlusal splint therapy remains limited, particularly regarding clearly defined endpoints and follow‑up timepoints.​

Therefore, this study aimed to compare the effectiveness of rehabilitation and occlusal splint therapies in patients treated at a medical center in Morocco. The primary endpoint was clinical remission, defined as the disappearance of the main clinical symptoms (joint noise, muscle and joint pain, and mandibular dyskinesia) assessed during a standardized clinical examination, evaluated at the follow‑up visit performed at least eight weeks after the end of treatment; we hypothesized that occlusal splint therapy would result in a higher clinical remission rate than rehabilitation.​​

## Materials and methods

This was a prospective longitudinal study conducted at the Department of Prosthodontics and Occlusodontics of the *Entre de Consultations et de Traitements Dentaires* (Dental Consultation and Treatment Center) in Casablanca, Morocco, over eight months from November 18, 2022, to July 9, 2023.​ The study was approved by the Pedagogic and Research Conduct Committee of the Faculty of Dental Medicine, Université Hassan II de Casablanca (approval number: FMDC-pr2c/01-2025). 

The study was conducted in accordance with the ethical principles for medical research involving human subjects outlined in the Declaration of Helsinki. Verbal informed consent was obtained from each patient prior to inclusion and before any study-related data were recorded. This was done for reasons of practicality and to reduce administrative formalities, while still adhering to ethical principles. All patients were fully informed orally about the objectives of the study, the nature of the clinical data collection, the proposed treatment modalities, and the implications, and their agreement was recorded in their medical file. This approach was approved by our institution's ethics committee, which deemed it appropriate for the context of this study. Patient confidentiality was preserved by anonymizing all clinical observation forms and removing any directly identifiable information from the database.​

Study population and sample size

Inclusion criteria comprised patients presenting with painful TMD symptoms, who provided informed consent, completed their treatment, and attended a follow-up visit at least eight weeks post treatment. Exclusion criteria were TMD of syndromic or systemic origin, ongoing orthodontic treatment, and any previous TMD treatment outside the prosthodontics/occlusodontics department.​

The study included 70 patients diagnosed with TMD. Among them, 44 patients underwent treatment with occlusal splints, while 26 received physiotherapy (rehabilitation). Regarding sample size, a formal a priori power analysis was not performed. Instead, a convenience sampling method was used, including all eligible patients who consulted the department during the study period. The final sample size was limited by organizational constraints, including the department's reception capacity, the clinical workload required for each case, and patient availability for long-term follow-up.​

Data collection tools and procedure

Data were gathered using a standardized clinical observation sheet (see Appendices) developed in collaboration with an epidemiologist to ensure comprehensive and clear data collection. Before the main study, this sheet was piloted over a three-day pre-survey phase to assess its clarity, acceptability, feasibility, and the approximate duration of administration. Based on feedback from this pilot phase, several items were reworded, and the final version of the sheet was refined, which supports its face and content validity in our clinical context.

The form included 14 items grouped into eight sections covering participant identification, consultation motives (joint noises, pain, dyskinesia), clinical examination findings, precise diagnosis (right, left, or bilateral disc displacement with reduction (DDWR) or disc displacement without reduction (DDWoR), treatment type (occlusal splint or physiotherapy), treatment duration, clinical evolution (favorable vs. failure), and follow-up status at least eight weeks after completion of therapy.​

Patient-reported items (e.g., presence of pain, joint noises, dyskinesia) were coded as binary variables (yes/no), whereas clinical examination findings and diagnosis were recorded by a single trained examiner. No composite numerical score or Likert-type scale was constructed; the sheet was designed for structured clinical documentation rather than for psychometric measurement.​

Interventions

Occlusal Splint Therapy

Occlusal splint therapy was delivered and monitored by supervising clinical staff. Splints were evaluated and adjusted approximately every two to three weeks. Patients were instructed to wear the splint daily throughout the day, except during meals, until clinical remission and during the subsequent eight-week follow-up period. Although some protocols recommend prolonged full-time wear, the wearing time was reduced as soon as symptoms resolved in order to limit potential occlusal adverse effects.​

Physiotherapy/Rehabilitation

Two rehabilitation modalities were employed: professional physiotherapy and self-directed “gymnotherapy.” For professional physiotherapy, patients were referred to a licensed physiotherapist, and a program consisting of 10 sessions targeting TMJ and cervical spine rehabilitation was prescribed. Exercises were initially performed under supervision at a frequency of two to three sessions per week, followed by daily home exercises over several weeks to months. Prescriptions could be renewed for extended corrective treatment when clinically indicated. Gymnotherapy comprised patient-performed exercises and techniques, including muscle stretching and strengthening, relaxation techniques, massage, and gentle jaw manipulation.​

Study conduct

Prior to the main study, a pre-survey was conducted over three days to train the investigator, test the observation form’s clarity, feasibility, and duration, and address potential issues. The observation form was refined based on feedback before launching the full study. Patients attended scheduled consultations primarily on Tuesday mornings. After informed consent, detailed clinical observations were recorded from the initial consultation through the treatment phase, which included either occlusal splint therapy or physiotherapy. Clinical data were collected at baseline (initial consultation), and treatment response was assessed at a follow-up visit performed at least eight weeks after completion of therapy to evaluate clinical remission, defined as the disappearance of joint noises, musculo-articular pain, and mandibular dyskinesia on standardized clinical examination. No additional standardized intermediate outcome assessment timepoints were predefined.​

Statistical analysis

Quantitative data were summarized using means and standard deviations (SDs), while qualitative variables were expressed as frequencies and percentages. The primary analysis compared the clinical remission rates between the occlusal splint and physiotherapy groups using the Chi-square test (or Fisher’s exact test where appropriate). To further explore the data, secondary bivariate analyses were conducted to evaluate the influence of age, sex, diagnosis (DDWR vs. DDWoR), and treatment duration on the outcome. All statistical tests were two-tailed, and p-values less than 0.05 were considered statistically significant. Data management and analysis were performed using standardized statistical software.

## Results

A total of 70 patients were included in the study, with ages ranging from 15 to 70 years and a mean age of 35.6 ± 14.9 years. The majority of the participants were female (82.9%, n = 58), while male patients represented 17.1% (n = 12). Regarding age distribution, 40% (n = 28) were under 30 years old, another 40% (n = 28) were between 30 and 50 years old, and 20% (n = 14) were over 50 years old.

The most common reasons for consultation were joint noise (70%, n = 49), followed by pain (68.6%, n = 48), and dyskinesia (65.7%, n = 46). These same clinical signs were frequently observed during the initial clinical examination, with identical proportions for joint noise (70%), pain (68.6%), and dyskinesia (65.7%). Diagnostic findings revealed that 30% of patients (n = 21) had bilateral DDWR, 25.7% (n = 18) had unilateral DDWoR, 22.9% (n = 16) had unilateral DDWR, and 21.4% (n = 15) had bilateral DDWoR.

Treatment was primarily of two types: 62.8% of patients (n = 44) were treated with occlusal splints, and 37.2% (n = 26) received specific physiotherapy targeting the TMJ. The mean treatment duration was 10.97 ± 7.23 months. Among all patients, 35.7% required a treatment period ranging from 6 to 12 months to achieve recovery.

Overall, a favorable outcome was observed in 84.3% of cases (n = 59), with full clinical recovery. Follow-up was conducted eight weeks after treatment completion in these 59 recovered patients, including 18 who had received physiotherapy and 41 who were treated with occlusal splints. A stable outcome, without recurrence, was observed in 100% of these patients. The remaining 11 patients were not included in the follow-up analysis as their treatment was still ongoing.

Analysis of factors influencing recovery showed that treatment type had a significant impact. Patients treated with occlusal splints had a recovery rate of 95.5%, significantly higher than the 65.4% observed in those who received physiotherapy (p = 0.001). Treatment duration also showed a trend: patients treated for less than six months had a recovery rate of 95.5%, compared to 84% for those treated for 6-12 months, and 73.9% for those treated for more than 12 months. However, this difference was not statistically significant (p = 0.139).

The type of diagnosis did not significantly influence the recovery rate, although patients with DDWR had a slightly higher rate (52.5%) compared to those with DDWoR (47.5%) (p = 0.583). Similarly, age and sex did not show statistically significant effects on recovery. Patients aged 30-50 had a recovery rate of 86.7%, those under 30 had a rate of 85.7% (p = 0.621), while female patients had a success rate of 86.2% versus 75% in male patients (p = 0.332) (Table 1).

**Table 1:** Summary of recovery and failure rates according to treatment and patient characteristics DDWR: disc displacement with reduction; DDWoR: disc displacement without reduction

Category	Sub-category	Recovery		Failure		P value
		Frequency	Percentage	Frequency	Percentage	
Treatment	Occlusal Splint	42	95.5%	2	4.5%	0.001
	Physiotherapy	17	65.4%	9	34.6%	
Treatment Duration	< 6 Months	21	95.5%	1	4.5%	0.139
	6 to 12 Months	21	84.0%	4	16.0%	
	> 12 Months	17	73.9%	6	26.1%	
Diagnosis	DDWR Bilateral	17	81.0%	4	19.0%	0.382
	DDWR Unilateral	14	87.5%	2	12.5%	
	DDWoR Unilateral	17	94.4%	1	5.6%	
	DDWoR Bilateral	11	73.3%	4	26.7%	
Age	< 30 Years	24	85.7%	4	14.3%	0.621
	30 to 50 Years	26	86.7%	4	13.3%	
	> 50 Years	9	75.0%	3	25.0%	
Gender	Female	50	82.2%	8	13.8%	0.332
	Male	9	75.0%	3	25.0%	

## Discussion

A clear female predominance was observed in our sample (82.9%), which aligns with previous research highlighting a higher prevalence of TMJ disorders among women. For instance, LeResche et al. [3] reported female-to-male ratios ranging from 1.4:1 to 2.6:1, while Pedroni et al. [4] found prevalence rates nearly four times higher in women. Similarly, Paulino et al. reported a 69% female distribution, and an intercontinental study involving over 85,000 adults showed that 62% of those affected were women [5]. Biological explanations commonly proposed include hormonal fluctuations, particularly in estrogen, and differences in pain perception and connective tissue. The role of estrogen, however, remains a subject of research. Landi et al. have suggested that estrogen could contribute to internal derangements of the TMJ [6].

The age distribution in this study, with a mean age of 35.6 years and 80% of patients being under 50, reflects the demographic most commonly affected by TMD. These findings are consistent with the literature, such as Poveda-Roda et al. [7], who reported a mean age of 40.5 years, and a Brazilian study showing a predominance of patients aged 20-49 [8]. This higher prevalence among young adults is often attributed to lifestyle factors, increased stress, and psychosocial influences, which have been identified as significant contributors to chronic orofacial pain by Wahlund et al. [9].

Symptomatically, joint noises (70%), pain (68.6%), and dyskinesia (65.7%) were commonly reported complaints, both in patient interviews and clinical exams. These findings match those of Di Paolo et al. [10], who found joint noises in 53% of patients, and Buescher [11], who noted that such noises are frequent diagnostic indicators, even if not always associated with functional limitations or pain.

Diagnostically, DDWR was slightly more frequent than DDWoR, accounting for 52.9% and 47.1% of cases, respectively. These diagnoses were confirmed via magnetic resonance imaging (MRI), improving diagnostic accuracy. DDWR typically manifests with joint clicking and mild discomfort, while DDWoR is more restrictive, with reduced mouth opening and deviation. These distributions align with the results of Poveda-Roda et al. [7] (DDWR in 44.8%, DDWoR in 6.5%) and are similarly reported by Wieckiewicz et al. [12] and Manfredini et al. [13]. The greater stability of DDWR and its lower risk of progression may explain its higher prevalence. 

In terms of treatment modalities, 62.8% of patients received occlusal splints and 37.2% underwent physiotherapy. The splints were adjusted every two to three weeks, with patients instructed to wear them continuously except during meals. Although some protocols suggest three to six months of use, caution is advised to avoid occlusal alterations. Physiotherapy included two approaches: professional physiotherapy (involving regular supervised sessions followed by self-guided exercises) and gymnotherapy, which focused on jaw muscle stretching, relaxation, massage, and joint mobilizations. Clinical data were collected at baseline (initial consultation), and treatment response was assessed at a follow-up visit performed at least eight weeks after completion of therapy to evaluate clinical remission, defined as the disappearance of symptoms on standardized clinical examination; no additional standardized intermediate outcome assessment timepoints were predefined.

Clinically, the results showed a significantly higher success rate with occlusal splints (95.5%) compared to physiotherapy (65.4%), with the difference being statistically significant (p = 0.001). These findings strongly suggest the superiority of splint therapy in our cohort. Similar results were reported by Wahlund et al. [9], who found that 62.1% of adolescents treated with occlusal splints were "completely well/very well improved" compared to only 17.9% in the physiotherapy group, a statistically significant difference (p < 0.001). Their findings, based on subjective reports, support the use of standardized clinical splint therapy as a more effective first-line treatment for adolescent TMJ pain. Notably, the study also emphasized the usefulness of switching to an alternative therapy for non-responders.

Treatment duration also appeared to influence outcomes. Patients who recovered within six months had a success rate of 95.5%, compared to 84% for those treated over 6-12 months, though this difference was not statistically significant (p = 0.139). These data suggest a trend favoring shorter treatment periods, though more research is needed to confirm whether early recovery is consistently linked to duration or simply reflects disease severity at baseline.

Supporting these findings, a systematic review published in 2023 examined 12 studies evaluating splint use in both DDWR and DDWoR [14]. All studies reported positive outcomes, though effectiveness varied. Statistical analysis revealed significant results in favor of splint therapy, with pooled odds ratios of 0.30 for DDWR and 0.36 for DDWoR. These consistent findings suggest that splints are a conservative treatment for internal TMJ disorders.

However, some studies present contrasting results. Shousha et al. conducted a comparative study on a therapy involving active jaw exercises, relaxation, and passive stretching (12 sessions of 15 minutes) versus full-time splint wear over six weeks [15]. They found that physiotherapy was more effective in reducing pain and improving mobility. However, this difference might reflect the short duration of follow-up, as splint therapy generally requires a longer period to yield its full therapeutic effect. Michelotti et al. similarly observed that physiotherapy tends to provide quicker symptomatic relief, while splints may be more beneficial over the long term [16].

The study by Van Grootel et al. highlighted significant differences in treatment modalities [17]. They reported that physiotherapy involves a significantly shorter total treatment time (by 10.4 weeks on average, p < 0.001), whereas splint therapy requires a significantly lower number of clinical visits to achieve success (7.1 fewer on average, p < 0.001). They concluded that physiotherapy might be preferable initially due to its shorter timeline, but splints remain advantageous in terms of reducing healthcare resource usage. These contrasting findings reinforce the importance of patient-specific treatment planning based on clinical presentation, lifestyle, and follow-up possibilities.

When comparing outcomes across diagnostic subgroups, no statistically significant difference was observed between DDWR (52.5% success) and DDWoR (47.5%) (p = 0.583). Greene et al. observed similar trends, noting that occlusal splints could reposition discs in DDWR cases but were generally ineffective for anterior disc displacement without reduction [18]. This highlights the importance of precise diagnostic classification in determining the suitability of splint therapy.

In terms of demographic factors, neither age nor sex showed a significant influence on treatment success. Patients aged 30-50 years showed a success rate of 86.7%, compared to 85.7% in those under 30 (p = 0.621), while females had a slightly higher success rate (86.2%) than males (75%) (p = 0.332). Though these differences were not statistically significant, the literature suggests that women may report more symptoms and seek treatment more often, possibly due to hormonal or psychosocial factors. Younger patients, owing to better tissue adaptability, may also respond more favorably to therapy. Still, such associations remain speculative and multifactorial, emphasizing the complex interplay between biological, psychological, and behavioral determinants of healing.

Limitations of the study

Despite the prospective design conducted in a real‑world clinical setting with standardized examinations performed by a single trained investigator (ensuring consistency), this study has several important limitations that should be emphasized. First, the sample size was relatively small (n = 70), mainly due to organizational constraints in the department (limited capacity and workload/time required for patient care), as well as patient refusal or non‑cooperation and follow‑up difficulties. Second, treatment allocation was nonrandomized and the groups were unequal (44 splint vs. 26 physiotherapy), which introduces a substantial risk of selection (allocation) bias; notably, our study reports that splint‑treated patients tended to return for completion of treatment and follow‑up, whereas some physiotherapy patients continued sessions outside the department and did not return once symptomatically improved, potentially inflating the observed success and follow‑up stability in one group.​

Third, outcomes were defined clinically as “recovery” based on the disappearance of joint noises, musculo‑articular pain, and dyskinesia on standardized clinical examination at a control visit ≥ 8 weeks after treatment completion; however, no validated patient‑reported outcome measures (e.g., pain scales, function or quality‑of‑life questionnaires) were used, and blinding of the examiner was not implemented, which increases the likelihood of measurement (information) bias. In addition, the study relied on patient reporting for some elements, and the authors identified potential “lying bias” and misunderstanding of questions, further supporting the possibility of information bias. Finally, because baseline symptom severity and other clinical or psychosocial confounders were not quantified using standardized scales and the analysis relied mainly on bivariate comparisons, residual confounding (e.g., patients with more severe symptoms preferentially receiving one modality) cannot be excluded and may partly explain the observed differences in recovery between treatments.​

Another limitation concerns the measurement tool used for data collection. Although the clinical observation sheet was developed with the support of an epidemiologist and piloted before the main survey to improve its clarity and completeness, it did not undergo formal psychometric validation (e.g., assessment of internal consistency, test-retest reliability, or construct validity), and no standardized scoring system was applied. The variables were mainly dichotomous (presence/absence of symptoms or signs), which may limit comparability with studies using validated TMD scales and may increase the risk of measurement and information bias.

## Conclusions

This study compared the clinical effectiveness of occlusal splint therapy and physiotherapy in the management of TMD. The findings demonstrated a significantly higher recovery rate with occlusal splints, but this should be interpreted as an association rather than definitive superiority because of the nonrandomized design and the risk of residual confounding and selection bias. However, physiotherapy also showed notable benefits, including faster symptom relief and a more comprehensive approach to functional rehabilitation. A combined therapeutic strategy may offer added value in certain cases.​ 

Despite its contributions, the study had several limitations, including a small sample size, unequal group distribution, limited follow-up from physiotherapy patients, potential reporting or comprehension biases, and non-randomization of allocation. These factors may affect the generalizability of the results.​ Overall, though, this research highlights the potential of conservative therapies in TMD management and underscores the need for further high-quality, long-term studies to refine clinical decision-making and promote evidence-based, individualized treatment plans. Future research should prioritize randomized controlled trials with better baseline characterization and validated outcome measures to reduce selection bias and confounding and to confirm comparative effectiveness over time.

## Appendices

Study questionnaire

Clinical Observation

Name of patient: ........................................................

Gender: .................................................................

Age: ..................................................................

Chief complaint

Noise: .................................................................

Pain: ..................................................................

Dyskinesia: .........................................................

Clinical examination

Noise: .................................................................

Pain: ..................................................................

Dyskinesia: .........................................................

Diagnosis

Right DDWR

Left DDWR

Bilateral DDWR

Right DDWoR

Left DDWoR

Bilateral DDWoR

Treatment 

Splint therapy

Physiotherapy

Recovery 

 Yes

 No

Duration of the Treatment

..................................................................

Follow-Up for 8 Weeks

Stability

Recurrence
